# What second-language speakers can tell us about pragmatic processing

**DOI:** 10.1371/journal.pone.0263724

**Published:** 2022-02-18

**Authors:** Ahmed Khorsheed, Sabariah Md. Rashid, Vahid Nimehchisalem, Lee Geok Imm, Jessica Price, Camilo R. Ronderos

**Affiliations:** 1 Department of English, Universiti Putra Malaysia, Serdang, Selangor, Malaysia; 2 School of Psychology, University of Nottingham Malaysia, Semenyih, Selangor, Malaysia; 3 Department of Philosophy, Classics, History of Art and Ideas, University of Oslo, Oslo, Norway; Potsdam University, GERMANY

## Abstract

Upon hearing the phrase *Some cats meow*, a listener might pragmatically infer that ‘Some but not all cats meow’. This is known as a scalar implicature and it often arises when a speaker produces a weak linguistic expression instead of a stronger one. Several L2 studies claim that pragmatic inferences are generated by default and their comprehension presents no challenges to L2 learners. However, the evidence obtained from these studies largely stems from offline-based tasks that provide limited information about how scalar implicatures are processed. This study investigated scalar implicature processing among L2 speakers of English and the degree to which differences in L2 proficiency and Theory of Mind abilities would modulate pragmatic responding. The experiment used an online sentence verification paradigm that required participants to judge, among multiple control items, the veracity of under-informative sentences, such as *Some cats are mammals*, and to respond as quickly as possible. A true response to this item is indicative of a logical *some and perhaps all* reading and a false response to a pragmatic *some but not all* reading. Our results showed evidence that scalar inferences are not generated by default. The answer linked to the pragmatic reading *some but not all* took significantly longer to make relative to the answer that relies on the logical interpretation *some and perhaps all*. This processing slowdown was also significantly larger among participants with lower English proficiency. Further exploratory analyses of participants’ Theory of Mind, as measured by the Social Skill subscale in the Autism Spectrum Quotient, revealed that socially inclined participants are more likely than the socially disinclined to derive a scalar inference. These results together provide new empirical insights into how L2 learners process scalar implicatures and thus implications for processing theories in experimental pragmatics and second language acquisition.

## Introduction

Paul Grice [1] proposed that human communication involves more than the mere encoding and decoding of messages. Critically, he argued that interlocutors often convey more than what their words literally mean through a variety of inferences. To make this concrete, imagine a professor who tells her students that *Some of you passed the exam*. Literally speaking, this means that at least one or two students passed the exam and even perhaps they all did. However, assuming she is speaking sincerely (i.e., she is not being playful), the students could surmise that she is being as informative as possible and, if all had indeed passed, she would have used the more informative quantifier *All*. The fact that she did not use *All*, her use of the weaker *Some* indicates that *not all* of them passed; thus, *some* implies *not all*. There are now many accounts for this sort of pragmatic inference, many of which rely on the notion of scales (*All* entails *Some*), which is why this inference is usually called a *scalar implicature*. This sort of pragmatic inference has been intensively studied for over two decades now and has led to much experimentation (e.g., [2–9]).

The modern outlook of psycholinguists about how scalar implicatures are processed in real time experiments has been later bridged by two main processing approaches that envisage two opposing views about the way in which scalar implicatures are computed; namely, the default theory [10] and Relevance theory [11]. According to the default theory, scalar inferences are generated by default without processing costs. For instance, upon hearing a sentence such as *Some parrots are birds*, the first interpretation that is immediately given rise to is the pragmatic interpretation, *some* [but not all]. The logical interpretation *some* [and possibly all] can arise only if the implicature is cancelled by contextual information. In cancellation, the processor must pass through a stage in which the upper-bound meaning is considered and then rejected based on implicit or explicit contextual factors. In such respects, the logical interpretation involves a two-step process, whereas the pragmatic interpretation involves only a one-step process [4] (see also [5] for an alternative explanation).

To the contrary of the default theory, Relevance theory holds that scalar inferences do not arrive automatically, but rather depend on the contextual situation [11]. In more technical terms, the pragmatic meaning arrives in a second-step process after the logical meaning was computed and then rejected for pragmatic purposes. As Bott and Noveck [4] propose, the explanations of these two accounts suggest two different predictions on the time course taken to process sentences with scalar terms. The default account predicts more processing times for logical meaning than the pragmatic meaning; and therefore, the time taken to process the logical meaning should be greater than or equal to pragmatic meaning. In contrast, Relevance theory assumes exactly the opposite: pragmatic meaning takes a longer time to process than the logical meaning because it is generated in a second step after the logical meaning was processed.

There are numerous processing studies that have investigated the predictions of these two theories in the last two decades but the evidence supporting them remains conflicting. Studies are mainly split into two camps: one is placing emphasis that scalar implicatures are generated automatically without processing costs (e.g., [7, 12, 13]), and the other is placing emphasis that scalar implicatures are late-arriving and they carry cognitive costs (e.g., [3–6, 14–16]). The underlying cause of this discrepancy is still lagely disputed, and the variable tendency with which some participants draw pragmatic inferences more than others adds another layer of complexity. More specifically, several reports suggest there is a substantial proportion of adults who tend to be more tolerant with their pragmatic violations than others (e.g., [4, 17, 18]). In reading a sentence such as *Some elephants are mammals*, people tend to vary on how they read the meaning of this under-informative sentence, that is, they either consistently interpret *some* as *not-all* or they they read *some* as *some*-*and-perhaps-all*. The underlying cause of this variability in interpreting scalar implicatures has been a subject of controversy [14, 18, 19], and it is undeniable that there are numerous subtle task- and item-related properties that may influence the tendency to derive pragmatic inferences, including stimuli type (i.e., universal vs. categorical sentences (e.g., [4, 19]), number of fillers in the experiment (e.g., [20]), number of response options (e.g., [21]), scalar diversity [22, 23]; and social contexts [24, 25] among others. However, more recent work has highlighted that this variation in comprehending pragmatic inferences can be largely rooted in individual differences in participant-internal cognitive characteristics, specifically Theory of Mind (ToM) (e.g., [26–28]).

ToM is thought to be involved in comprehending different types of pragmatic inferences, among which is the computation of scalar implicatures [29]. To engage in ToM requires the interlocutor not only to interpret the linguistic behavior from their own self-perspective, but also from the perspective of others and therefore to keep track of the interlocutor’s epistemic state necessary for scalar implicature derivation [1, 11, 30, 31]. Some studies suggest that switching from a default self-perspective to someone else’s perspective is accompanied by an effortful process [32, 33]. It is effortful because holding in mind information about someone’s belief and (possibly) coordinating it with other linguistic and non-linguistic cues in order to inform a subsequent judgment does incur processing costs [32, 34]. ToM and executive functions (EF) are often correlated with one another [35, 36], and therefore the decision to reject a pragmatic violation could be a product of two interrelated processes: the involvement of working memory capacity and the application of ToM in inferring the speaker’s state of knowledge [18]. But how can ToM account for the behavioral variation in interpreting pragmatic inferences?

Recent experimental investigations on the role of ToM in determining pragmatic behavior show that participants’ variable performance in comprehending scalar inferences is largely correlated with participant’s social skills or *mindreading abilities* (e.g., [17, 25, 37]). Those with inferior ToM abilities tend to be more literal in interpreting under-informative sentences than those with strong ToM abilities, in both irony [37] and scalar implicatures [29, 38]. For instance, Fairchild and Papafragou [29] examined the role of ToM in scalar implicature computation, particularly the unique roles of EF and ToM abilities in deriving scalar inferences. They used the digit span task and high cognitive load memory trials embedded in a scalar implicature task to measure EF, whereas the participants’ performance on the mind in the eyes task and strange stories was used to provide a measure of ToM abilities. Fairchild and Papafragou found that both EF and ToM are significantly and positively correlated with the rate of pragmatic interpretations; however, when the shared variance between the EF and ToM was controlled for, only ToM was a significant predictor of the variance in pragmatic responding. The participants who had stronger ToM abilities tended to draw the pragmatic reading of scalar implicatures more often. Importantly, this evidence corroborates previous reports arguing about the impact of ToM in irony comprehension by Spotorno and Noveck [37]. They found that socially-inclined individuals, as measured by Social Skill subscale in Baron-Cohen’s Autism Quotient (AQ) questionnaire, are more likely to discern irony compared to socially-disinclined participants. Their study proposed that one’s engagement in ToM reasoning plays a significant modulating role in pragmatic comprehension.

Given that ToM itself can explain some the inter-individual variability in interpreting pragmatic inferences [29, 37], the question of how L2 learners use their ToM abilities to calculate a pragmatic inference remains under-explored. Several developmental studies on children showed evidence of a strong bidirectional relationship between ToM abilities and linguistic competence (e.g., [39, 40]), but we have limited information about how L2 adults with varying proficiency levels would depend on their ToM to compute scalar inferences. This being so, one may reasonably hypothesize that those with stronger linguistic skills are more likely to have better ToM abilities and might therefore have an advantage in drawing scalar inferences. Nevertheless, this proposition remains hypothetical and lacks supporting evidence.

## Scalar implicature in L2 context

The empirical evidence about how L2 speakers process pragmatic inferences is limited and relatively under-explored (see [41] for a comprehensive review). When discussing scalar implicature comprehension in L2 context, Slabakova [42] was the first to directly address implicature understanding among L2 learners. Slabakova tested the extent to which Korean and English L1 speakers and Korean L2 learners with different levels of English proficiency would be sensitive to under-informative sentences in two main experiments: contextualized vs non-contextualized. The contextualized experiment included under-informative sentences that were provided with a context to enrich the pragmatic meaning, whereas the non-contextualized experiment comprised under-informative sentences to which the participants have to evaluate and judge their truth-value without a context. Slabakova found evidence that the two proficiency groups (i.e., intermediate vs. advanced) were similar in their pragmatic performance and they rejected under-informative sentences more often than the two L1 speaker groups, in both the contextualized and non-contextualized experiment. Critically, this finding was at odds with the general view that L2 learners have less processing abilities and they are less accurate than L1 speakers (see [43, 44] for review), but Slabakova proposed that L2 learners, due to their enhanced executive functioning and attentional abilities, can exhibit better responses on metalinguistic tasks. Slabakova concluded that generating scalar inferences is an automatic cognitive process and inferring the pragmatic meaning is therefore not problematic to L2 learners regardless of proficiency level (see also [45–47]).

Inspired by Slabakova’s [42] findings, Dupuy and colleagues [47] recently compared the performance of French monolinguals to French bilingual adults (upper-intermediate). The French bilinguals were simultaneously tested in their L1 and L2; and therefore, they took half of the stimuli in their L1 and the other half in their L2 in a within-subject design. Their results revealed that French L2 learners had a higher rate of pragmatic interpretations than that of French monolinguals, but the bilingual participants had an identical proportion of pragmatic responses in their L1 and in L2. Dupuy et al. explained their results based on the assumption that having to switch between two languages made the participants more aware of pragmatic cues and hence more enhanced pragmatic abilities. In a follow-up experiment, Dupuy et al. made amendments and tested scalar implicature comprehension in a between subject-design. Their results revealed that L2 learners behaved much like L1 speakers, giving a comparable proportion of pragmatic interpretations to scalar implicatures. Dupuy et al. suggested that Slabakova’s explanation of the assumption that L2 learners tend to derive more pragmatic interpretations than monolinguals is unsupported, and thus learning a second language or being a bilingual does not seem to enhance pragmatic abilities among L2 learners.

However, opposed to Dupuy et al.’s view, there are several reports which suggest that bilingualism enhances pragmatic ability (e.g., [48–50]). More specifically, bilingualism enhances the executive control system (e.g., [51–55]), and this enhanced exective control function is thought to assist bilinguals to have much easier access to implicature derivation than L1 monolinguals. Siegal et al. [48] tested whether bilingualism would confer an advantage in the ability to draw scalar implicatures among children aged 4 to 6 who were either monolingual in English or Japanese or bilingual in the two languages. The monolingual participants were given the test material in their respective L1 languages and only in Japanese to the bilingual children. All groups were told a story in which a teddy bear was very good at putting hoops on a pole. They subsequently saw a picture showing that the bear and hoops were all on the pole. Then a puppet described that event by producing an under-informative sentence such as “Some of the hoops on the pole” and the children were asked to evaluate the appropriateness of the description that the puppet gave of the event. Siegal et al. [49] found evidence that bilingual children, despite their lower vocabulary scores, were more advanced than their monolingual peers in showing sensitivity to scalar implicatures. Siegal and colleagues suggested that the source of bilinguals’ pragmatic superiority compared to L1 speakers is not linguistic. However, bilingual children’s precocious pragmatic development is possibly due to their enhanced executive control skills, or maybe these bilingual children develop “heightened pragmatic skills as a compensation of their weaker knowledge of core language” [48].

It is worth noting that these conclusions made by Siegal et al. do not seem to corroborate recent results reported by Antoniou and Katsos [56]. Antoniou and Katsos also used a large battery of pragmatic tests that probe into the knowledge of different types of conversational implicature. They used two tasks on scalar implicature comprehension and the maxim of quantity (see Antoniou and Katsos [56] for a detailed description of the tasks). Their study compared the performance of three groups of participants whose years of age range between 6 and 9: (i) monolinguals, (ii) bilectals, and (iii) and multilinguals. Antoniou and Katsos [56] found no statistical difference between bilingual, bilectal, and multilingual children’s understanding skills. Multingual and bilectal children exhibited monolingual-like understanding of implicatures; and therefore, there was no multilingual-exclusive pragmatic advantage revealed, consistent with a recent study exploring the pragmatic abilities of Slovenian monolingual and Slovenian–Italian bilingual 10-year-olds [47]. Their results also did not support an effect of executive functioning on children’s pragmatic ability. However, participants’ proficiency in the language of testing as well as years of age were critical predictors of implicature understanding. Antoniou and Katsos [56] suggested that implicature understanding is a “pragmatic–communicative skill that largely depends on children’s language abilities.”

All in all, these previous reports show that the empirical landscape on how bilinguals compute scalar inferences remains less clear in the literature, specifically whether the superior pragmatic ability among L2 individuals was due to the automatic processes involved in generating the implicature, to a bilingualism advantage, to linguistic proficiency, or to a methodological bias as the evidence supporting previous claims comes almost exclusively from offline-based tasks. Recent studies on second language sentence processing demonstrate a significant difference in L2 performance across online and offline tasks [57–60]. The present study aims to address this issue by investigating scalar implicature comprehension among L2 adults using an online approach. Two groups varying in their L2 proficiency (i.e., “low” vs “high”) were recruited. Our prediction is this: if scalar implicature computation is default-based, then the *low* and *high* English proficiency participants should derive comparable proportions of pragmatic interpretations to scalar implicatures, and to have indistinguishable processing times for their pragmatic and logical interpretations. However, if the inferential process is cognitively effortful and mediated by proficiency level, then we expect to have larger pragmatic processing delays and inferior pragmatic performance by those participants with low English proficiency relative to the highly proficient participants. Further, the study also investigates, on an exploratory basis, the degree to which ToM reasoning abilities interact with implicature derivation and proficiency level, in relation to the matter discussed in the previous section.

## Experiment

### Participants

The participants were 213 L1 Bahasa Malay speakers of English from Universiti Putra Malaysia (UPM). All were undergraduates and their English proficiency was categorized into two levels: 116 with *low* English proficiency (age range 18–26, *M* = 22.06, *SD* = 1.8, all females) and 97 with *high* English proficiency (age range 18–26, *M* = 22.2, *SD* = 1.4, all females). The level of proficiency was determined based on the results obtained from the Malaysian University English Test (MUET), Band 3 and Band 4 respectively. MUET is a localized standard measure of English proficiency utilized by the Malaysian universities for students’ admission. The MUET comprises a six-band scale, Band 1 to Band 6. According to the Malaysian Examination Council, students with MUET band 3 are equivalent to those with IELTS band 5, whereas the students with MUET band 4 are equivalent to those with IELTS band 6. For the purpose of this study, the participants who obtained band 3 in their MUET were described as ‘*low* proficiency’ users of English, whereas those who obtained band 4 were described as ‘*high* proficiency’ users of English.

### Materials

The study used (i) a truth-value judgment task to examine the extent to which participants are sensitive to under-informative sentences, and (ii) the Autism Spectrum Quotient [61] to examine how individual differences on the Social Skill subscale would influence the rate of pragmatic interpretations of scalar implicatures. As additional exploratory measures, we also collected data from participants using the Systemizing Questionnaire (SQ-R, [62]) and Big Five Inventory (B5, [63]). Greater details on these instruments are given in the following subsections.

#### Truth-value judgment task

This is the pragmatic task that assessed the extent to which the participants would derive the pragmatic meaning embedded in under-informative sentences. In this task, participants read sentences and provided absolute truth-value judgments about them using “True” and “False” responses. The task was based on Bott and Noveck’s Experiment 3 [4], and consisted of evaluating 6 types of sentences expressing different categorical relationships (see Table 1). The sentences that are labeled as T1 are the *experimental* sentences that give rise to a scalar implicature, whereas T2 to T6 serve as controls. There was a total of 54 items in the task.

**Table 1:** Examples of test sentences.

Reference	Example sentence	Appropriate response
T1	Some parrots are birds	?
T2	Some birds are parrots	True
T3	Some parrots are fish	False
T4	All parrots are birds	True
T5	All birds are parrots	False
T6	All parrots are fish	False

*Note*. T1 sentence is labeled with a question mark because it can be considered false and sometimes true–this depends on whether the participant draws the inference or not.

Similar to Rips [64] and Bott and Noveck [4], T1, T2 and T3 are statements carrying the same quantifier *some* but have different category constructions. For instance, T1 and T3 are a case in which the exemplar is the subject of the sentence and the category is the predicate, whereas T2 is a case in which the category is the subject and the exemplar is the predicate. The sentences T4, T5 and T6 have equivalent constructions to that in T1, T2 and T3 respectively, but they employ the quantifier *all*. T1 sentences are the only sentences that are associated with implicatures and can therefore be true or false depending on whether the participant draws the inference or not, whereas T2 to T6 are only control sentences that are either patently true or patently false.

It is worth noting that the exemplars and their categories in our study were developed to fit the background knowledge of our Malay participants. One reason for this is the fact that the exemplars used in previous studies may not fit the participants of the present study. For instance, in a sentence such as *Some haddock are fish* from Bott and Noveck, the exemplar word *haddock* may not be known to the participants as an exemplar word that belongs to the *Fish* family, or this exemplar is possibly not existent in their world knowledge or L2 knowledge. Thus, we found that planning a new material was deemed necessary. A total of 35 L1 Malay undergraduates (who did not take part in the main experiment) were recruited to participate in a categorization task. They were given a sheet in which there were six categories presented in six columns. They were also briefed and instructed to list as many representative exemplars of the suggested categories as possible and based on their responses, 9 exemplars from each category were selected to be included in the test materials (see S1 Appendix).

The study also made sure that the quantifier *some* in English has its equivalent lexical item in Malay language to avoid any bias associated with participants’ perception of the scalar term *Some*. The Malay language has the quantifier *Sesetengah* which is equivalent to the quantifier *Some* in English. To illustrate, consider the following example:

1) *Sesetengah nyamuk ialah seranggah*
 ‘Some mosquitos are insects’
  −> Some but not all mosquitoes are insects.

The Malay quantifier *Sesetengah* can denote either singular or plural entities. It has a general existential meaning as in the English translation and can trigger the some-but-not-all implicature. *Sesetengah* is also similar to the English quantifier *Some* in its semantic and syntactic realization, and thus we consider *Sesetengah* and *Some* to be close equivalents.

#### Exploratory measures: Autism Spectrum Quotient (AQ), Systemizing Quotient-Revised (SQ-R) and Big Five Inventory (B5)

As exploratory measures, we collected additional personality measures from participants using the AQ [61], SQ-R [62], and B5 [63]. The AQ is a self-administered tool that measures the degree to which adults show autism-like traits in their everyday behavior [61]. It comprises 50 questions that assess five key areas: communication, social skill, attention switching, attention to detail, and imagination. Adults with autistic-like behavior are thought to show either mildly or strongly poor communication skills, poor social skills, poor imagination, strong attention to detail, and poor attention-switching. The present study used this questionnaire to assess the extent to which individual differences on the Social-Skill subscale would modulate the tendency to derive pragmatic interpretations. As previously mentioned, the Social-Skill subscale was used as a reflective measure of the ToM abilities involved in irony and implicature comprehension [37]. Socially-inclined individuals tend to calculate pragmatic inferences more than the socially-disinclined.

The SQ-R is also a self-administered questionnaire that measures individual differences in systemizing, i.e., the extent to which one can analyze systems, extract rules, and predict outputs [62]. According to Baron-Cohen [62, 65], systemizing individuals are associated with attention to detail and the seeking of exact truth. The SQ-R has 75 items covering areas known to be associated with social, domestic, mechanical and abstract systems. This study used the SQ-R to measure participants’ systemizing skills and find out how individual differences in systemizing are likely to be linked to participants’ (in)tolerance of pragmatic violations. As suggested by van Tiel and Schaeken [66], hearers may base their judgments on “statistical patterns” that help them gauge the likelihood that a potential interpretation is relevant to the speaker’s intended meaning (see also Barbet and Thierry [19] for relevant evidence).

The B5 questionnaire comprises 50 questions assessing 5 dimensions of personality that cover extraversion, agreeableness, conscientiousness, neuroticism, and openness. *Extroversion* covers facets that pertain to one’s being energetic, sociable, adventurous, and enthusiastic; *Agreeableness* refers to attributes of being sympathetic, helpful, forgiving, and cooperative; *Conscientiousness* includes characteristics such as being organized, thorough, cautious, and responsible; *Neuroticism* refers to characteristics such as one getting easily anxious, tense, being unstable, and emotional; and *Openness* to traits such as being imaginative, intelligent, artistic, and curious. We used B5 to examine whether or not different participant-specific traits may influence the tendency to derive implicatures [14, 18]. Taken together, this makes a total of 11 personality characteristics that we included in this study to examine the effect of individual differences on scalar implicature derivation.

### Data collection procedure

**Fig 1:**
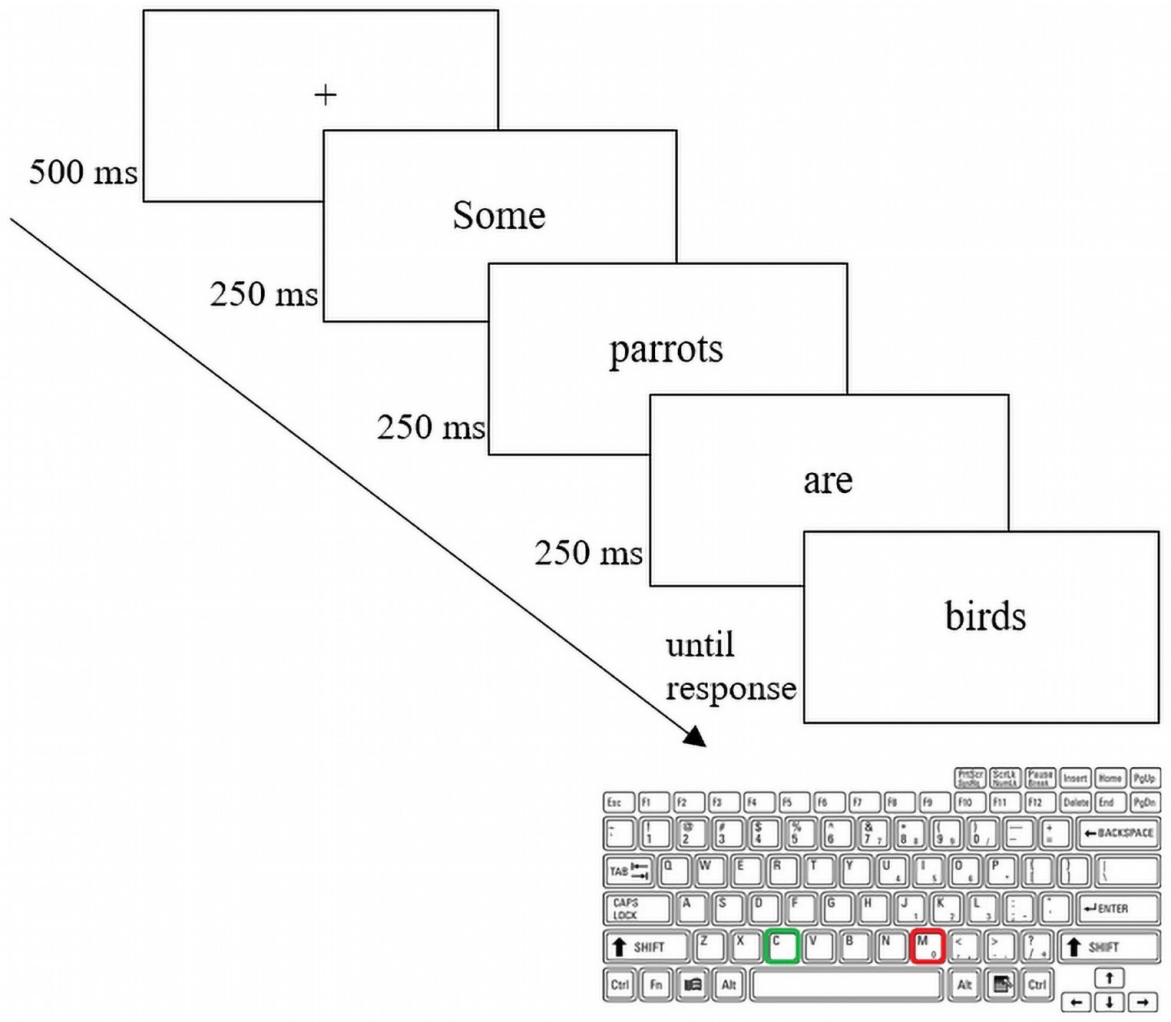
An example of the display of the test sentences including a fixation cross, series of words making up a categorical sentence, and a response keyboard.

The tasks were administered in the following order: the truth-value judgment task, the AQ, the SQ-R, and the B5, respectively. In the truth-value judgment task, the participants were placed in front of a computer and were instructed to judge the veracity of categorical sentences by using keyboard buttons labeled True and False, “c” and “m” on a QWERTY keyboard. The task included 54 trials. Each trial in the experiment consisted of a fixation cross and then a presentation to the sentence in the form of words appearing in sequence. The fixation cross remained on the screen for 500msec and then was replaced by the sentence words that were consecutively flashed onto the screen, one word at a time. Each word remained on the screen for 250msecs, with a gap of 50msec between each word presented (see Fig 1).

At the end of the sentence presentation (i.e., sentence reading), the participants were required to evaluate the truth value of their reading and judge as quickly as possible by pressing the green button labeled “True” if they thought the sentence was true or by pressing the red button labeled “False” if they thought the sentence was false. Rejecting T1 sentences corresponds to a pragmatic reading of the under-informative sentences, whereas accepting T1 sentences corresponds to a logical interpretation, which would indicate that the participants were not aware of the infelicity of under-informative statements.

Before starting the experiment, all the participants had a practice session. They saw 18 practice trials with exemplars and categories that were not part of the stimuli in the main experiment. The practice trials also followed the same presentation procedure of the trials in the real experiment and the participants were encouraged to ask questions during the practice session in order to work independently during the experimental session. The experimental session was divided into three blocks in order to give the participants two brief pauses. The participants also saw one dummy sentence at the beginning of each block to avoid problems associated with starting the experimental phase. A Latin Square design was used to create six lists of sentences so that every exemplar from a category was used only once per list. All participants’ responses and reaction times were recorded using E-prime software (version 3.0). They took an average of 45 minutes to complete the entire experiment.

### Ethics statement

The participants were informed that this research was approved by the UPM Research Ethics Committee (reference number: JKEUPM-2018-197). All participants received a cash payment worth $2.50 upon task completion.

## Analysis and results

### Data handling and exclusion criteria

All data was analyzed using R [67] and the lme4 package for fitting (generalized) linear regression models [68]. Prior to our main analysis, we removed data from participants who scored less than 70% accuracy on all control conditions (T2-T6). This led to the exclusion of 3 participants, leaving the total number of participants at 210. For our analysis of reaction times, we removed all incorrect responses in the control conditions. We also discarded responses shorter than 400 milliseconds and longer than 6 seconds, which resulted in the exclusion of less than 3% of the data. This choice of outlier removal did not affect the interpretation of our results. The remaining reaction time data was then log-transformed, since the residuals of a simple intercepts-only regression model were not normally distributed, violating the assumptions of the linear model. We chose the log-transformation following the results of a Box-Cox test [69]. The data and analysis script are freely available as part of the S1 Appendix. All details on each of the statistical models can be found in the supplied R script.

Our analysis was divided in two parts, corresponding to the twofold goal of our study. First, we examined the explicit role of proficiency in scalar implicature comprehension and how our L2 processing time data would reconcile with the theoretical predictions of the default theory and Relevance theory as discussed in the Introduction. Subsequent to this, we carried out exploratory analyses on possible sources of individual variation in pragmatic understanding as measured by the AQ, SQ-R and B5 questionnaires.

To analyze our data, we fitted regression models to the forced-choice data and the reaction times using Condition (T1-T6) and Proficiency (high vs. low) as independent variables. We additionally included the trial number as a control variable for the reaction time analysis. An important characteristic of regression models is that they are robust at handling missing values. This means that, in contrast to the procedure in Experiment 3 of Bott and Noveck [4], we no longer need to remove those participants who are consistently logical or consistently pragmatic. Thus, all data points from all 210 participants were included in our analyses. P-values were computed via model comparison.

### Implicature derivation rate

**Fig 2:**
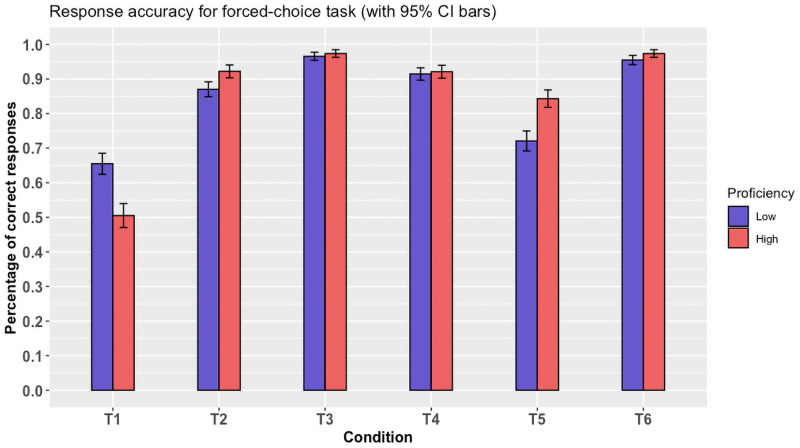
Choice proportions as a function of condition (T1-T6) and proficiency (low vs. high). Note that for the T1 condition, pragmatic responses were coded as ‘0’ and logical responses as ‘1’.

The forced-choice data was analyzed using mixed-effects, logistic regression. Before inferential analysis, we carried out descriptive analyses and the results showed that all participants were accurate above chance in all control conditions (T2-T6). In the critical T1 condition, the participants in the *high* proficiency group were equivocal between logical and pragmatic interpretations (50.5% logical responses), whereas those in the *low* proficiency group seemed to overall prefer a logical interpretation over a pragmatic one (65.4% logical responses). This can be seen in Fig 2 below.

For the inferential analysis, we first fitted a model including Proficiency (levels: ‘high’ and ‘low’) and Condition (T1-T6) as fixed effects as well as their interactions. We added random intercepts by subject and by item, as well as random slopes by item for the factor Proficiency. This was the maximally converging model, following the recommendations of Barr et al. [70]. The factor Proficiency was sum-contrast coded, while the factor Condition used default contrast coding. This first model showed a significant difference between T1 responses and all other conditions (T2-T6) regardless of proficiency level (all *z*-values >10 all *p*-values < 0.001). It also showed that there is a main effect of proficiency (*z* = 5.8, *p* < 0.001), with more proficient participants having higher accuracy on average (85.6%) than those in the low proficiency group (84.6%). This effect is seemingly driven by the responses in the T2 and T5 conditions.

To examine the differences between proficiency groups more closely, we followed-up this model with one that examined only T1 responses and coded Proficiency using treatment contrast coding. This model showed a significant difference between proficiency groups (i.e., low vs. high), confirming that participants in the high proficiency group behaved more pragmatically than those in the low proficiency group (*z* = 2.9, *p* < 0.005).

### Implicature processing time

**Fig 3:**
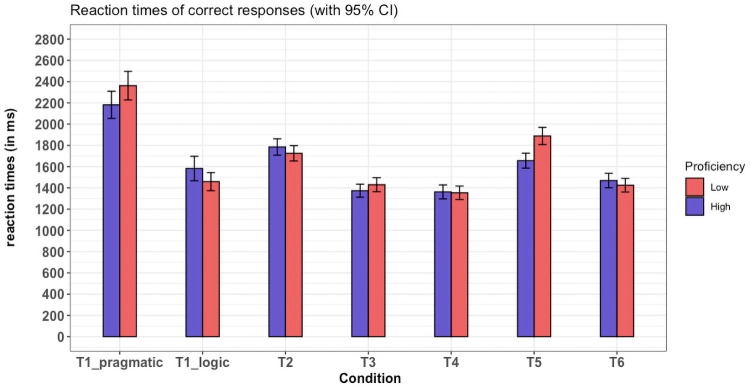
The reaction times as a function of condition (T1-T6) and proficiency (low vs. high). Error bars show confidence intervals.

To analyze reaction times, we fitted linear, mixed-effects regression models to the log-transformed response times. Prior to the analysis, we split the responses made to T1 sentences into ‘T1_logic’ and ‘T1_pragmatic’, depending on whether the T1 sentence was accepted or rejected, respectively. This division allows a within-subject measure of the change in reaction time when the participant makes a logical response or a pragmatic response (see also Bott and Noveck [4]). All regression models included the maximal random-effects structure granted by our experimental design (see Barr et al. [70]), namely; random intercepts by items and participants, as well as random slopes for both main effects and their interaction by items and random slopes for the factor ‘Condition’ by participants. Both factors were coded using treatment contrast coding. The average reaction times for all conditions (T1-T6) are shown in Fig 3 below.

As shown in Fig 3, our experiment broadly replicates the results of Bott and Noveck’s Experiment 3: Participants took on average longer to reject the T1 statements (i.e., understand them pragmatically) than to correctly reject statements in each of the control conditions that required a FALSE response (T3, T5 and T6, all *t*-values > 8, all *p*-values < 0.01). Further, the participants did not take a significantly different amount of time to accept statements in the T1 condition (i.e., understand them logically) than to correctly accept statements in the control conditions that required a TRUE response (T2 and T4 combined). When seen individually, response times to T1_logic were wedged between the control conditions, with significantly shorter response times to T2 and significantly longer response times to T4. These results suggest that, on average, L2 speakers of English show a similar processing cost of understanding scalar implicatures relative to L1 speakers reported in Bott and Noveck [4].

Crucially, our results show evidence that scalar implicature computation is mediated by Proficiency among our L2 participants. There was a significant interaction effect between the two T1 conditions (i.e., T1_logic and T1_pragmatic) and Proficiency (*t* = 2.2, *p* < 0.05), suggesting that the processing effort of pragmatic interpretations was affected differently by level of proficiency: participants in the low proficiency group took numerically longer than those in the high proficiency group to process a pragmatic interpretation of T1 sentences (2362 ms in the low proficiency group vs. 2181 ms in the high proficiency group), but not a logical interpretation (1458 ms in the low proficiency group vs. 1582 ms in the high proficiency group). This effect was not visible for the interaction of either of the two T1 interpretations and their respective control sentences (T3, T5, and T6 for T1_pragmatic and T2 & T4 for T1_logic, all *p*-values > 0.05). Re-running the same model but coding Proficiency using sum contrast coding showed that there was no an overall, main effect of Proficiency on processing time (*t*-value = 0.575, p > 0.05).

### Individual differences in scalar implicature comprehension: Exploratory analyses

As mentioned in the Introduction, there is uncertainty in the literature about the cause of individual differences in comprehending scalar inferences. With this in mind, we are interested in exploring the degree to which individual variation among L2 speakers could account for any further comprehension differences above and beyond the effect of proficiency. The first and most important source of potential individual variation to be considered is that relating to a comprehender’s social skill. The Social-skill subscale in Baron-Cohen’s AQ was previously taken as a measure of ToM abilities [37]. Fairchild and Papafragou also showed that participants with inferior ToM reasoning abilities are more likely to be literal in their interpretation of scalar implicatures compared to others with strong ToM abilities [29]. Therefore, we fitted a binomial logistic model to analyze the influence of scores on the AQ Social-skill subscale on the implicature derivation rate. This model included the by-participant average rating elicited by the AQ questionnaire on the areas of communication, social skill, attention switching, attention to detail, and imagination. We also included the by-participant average rating collected from the SQ-R and B5 questionnaires. This was performed to explore the effect of the Social-skill subset while controlling for multiple other potential sources of individual variation.

For simplicity, we fitted the model only on the comprehension data for the critical T1 condition. We included all new variables as fixed effects and as random slopes by items in the analysis. Given the exploratory nature of this analysis, we corrected for multiple comparisons using a Bonferroni correction. This meant dividing the value of our alpha threshold by 21, i.e., the number of total comparisons (11 main effects plus 10 potential interactions). This left us with a new alpha threshold of 0.002. With this fairly conservative measure, our model suggests that only 2 of the tested variables affect the derivation rate: Social-skill subscale (*z* = 4.87, *p* < 0.001), and Imagination subscale (*z* = 4.89, *p* < 0.001). Both measures are part of the AQ inventory.

**Fig 4:**
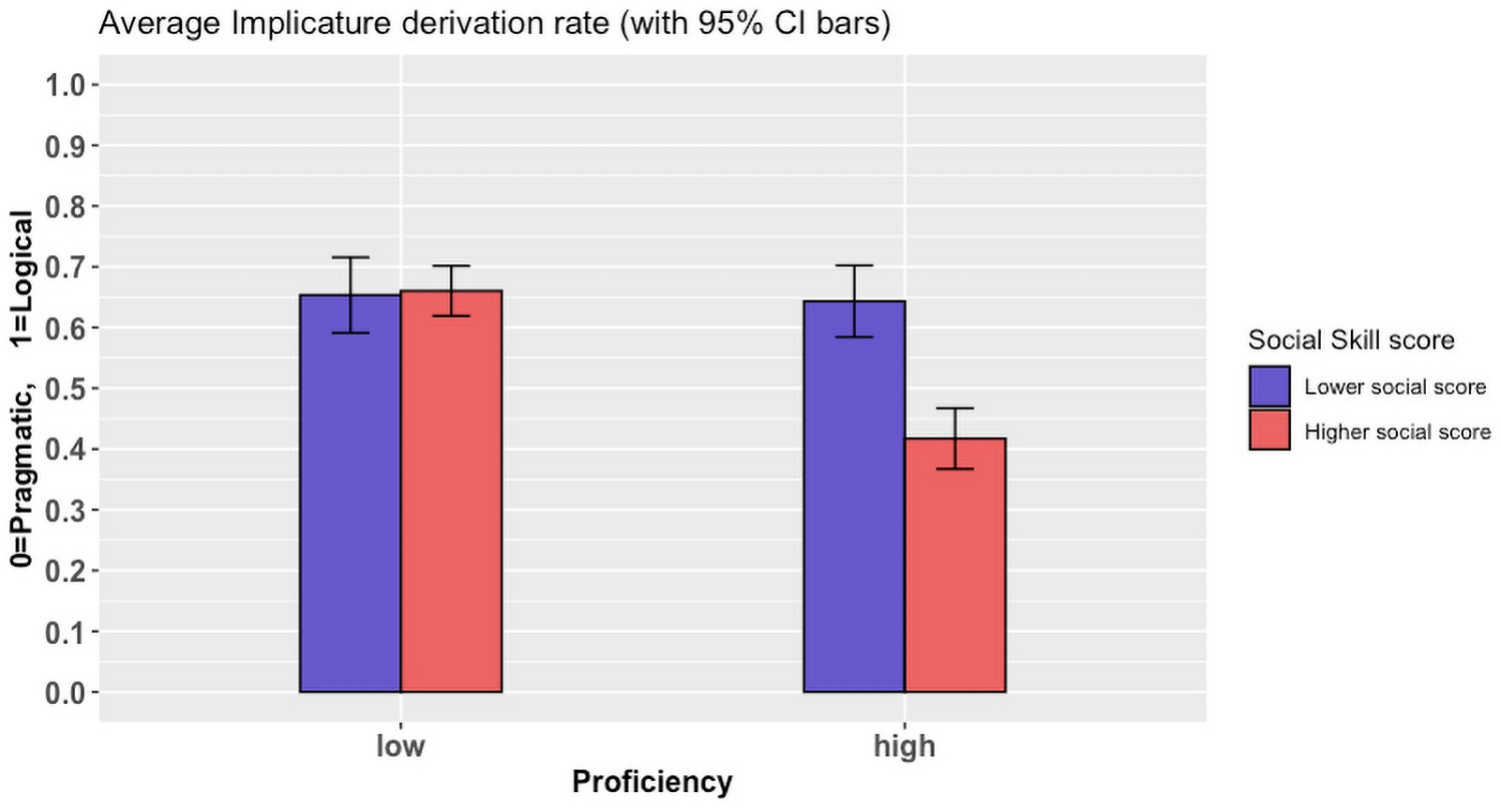
Interaction between proficiency and social-skill on the derivation of scalar inferences. Social-skill is dichotomized for illustration purposes only.

We followed-up on this finding by considering the potential interaction of each of these two variables with Proficiency by fitting two separate models for each variable. After controlling for the effect of imagination, only the model for Social-skill showed a significant interaction with implicature derivation rate after the Bonferroni correction (*z* = 3.986, *p* < 0.001). Participants with better social skills (as indicated by lower scores on the Social-skill subscale) were more likely to derive a pragmatic interpretation of our critical T1 sentences than those with poor social skills. This effect was particularly visible for participants with higher language proficiency, although the interaction between Social-skill and Proficiency did not meet our strict significance threshold (z = 2.648, *p* = 0.008). Fig 4 below illustrates this result. Note that the Social-skill was measured and analyzed as an ordinal variable from 1–10 (with lower scores denoting better social skills and vice versa). It is dichotomized here for the purpose of illustration only.

This finding of the effect of social skill on implicature derivation is in line with recent work on the relationship between Theory of Mind and scalar implicature computation (see Fairchild and Papafragou [29]). It also adds to the literature the possibility that language proficiency may interact with social skills among L2 speakers. However, this evidence is exploratory in nature and we thus leave it open for future research to test this interaction in a confirmatory paradigm.

## Discussion

Our study investigated scalar implicature processing among L2 speakers and the extent to which individual differences in language proficiency and Theory of Mind abilities would modulate the tendency to calculate pragmatic inferences. Previous studies on scalar implicatures in L2 context suggest that pragmatic inferences are generated by default and their comprehension presents no challenges to L2 learners regardless of proficiency level (e.g., [42, 45–47]). However, the evidence obtained from these studies remains inconclusive as the results almost come exclusively from offline paper-based tasks that provide little-to-no information about how scalar inferences are processed prior to their manifestation in the form of a response. To fill in the gap, we conducted an online experiment that measures the time taken to compute the pragmatic meaning relative to the literal meaning of scalar implicatures. Our paradigm was closely based on Bott and Noveck’s influential study that required participants to read under-informative sentences such as “Some eagles are birds” and judge their truth-value using a True or False press button. Participants’ rejections to under-informative sentences meant that the participants are able to read the pragmatic meaning *some-but-not-all* embedded in the quantifier *some*, whereas participants’ acceptances to these under-informative sentences meant that the participants are not sensitive to the pragmatic meaning and hence they treated *some* in its literal sense, as *some-and-possibly-all*.

Our study was guided by the predictions of two theoretical accounts which envisage opposing views on how logical and pragmatic interpretations unfold: the default theory [10] and Relevance theory [11]. While the default theory predicts longer processing times for the logical meaning than pragmatic meaning, Relevance theory postulates the opposite (see also Bott and Noveck [4] for alternative explanations). Our prediction was this: if the pragmatic meaning is truly generated by default, then we should observe no meaningful processing differences between the pragmatic and logical interpretations made to under-informative sentences with *Some*, and correspondingly no significant differences in the rate of their pragmatic interpretations between participants with low and high English proficiency. However, if generating scalar inferences is an effortful process that arrives in a secondary step, we should find significant processing slowdowns in the pragmatic responses relative to logical responses. What’s more, the proportion of pragmatic responses made to under-informative sentences should vary as a function of language proficiency.

Critically, our results provided evidence that scalar inferences are late-arriving and they carry cognitive costs. The two proficiency groups exhibited significant processing delays in interpreting the critical word ‘some’ as *some-but-not-all* relative to the interpretation *some-and-possibly-all*. Importantly, the pattern of response times differed between proficiency groups, suggesting that computing pragmatic inferences is more cognitively challenging to participants with lower English proficiency. Similar to Bott and Noveck [4], we carried out tests that compared the control items to T1-pragmatic and the results revealed that a “false” response to T1 sentences was significantly slower than the responses made to control sentences in the design, including T3 and T5 that requires a “false” response too, and thus evidence denoting that processing T1-pragmatic interpretations is characteristically different from processing literal interpretations.

Our results are at odds with the proposition that scalar inferences are generated by default among L2 learners (e.g., [42, 45, 47]). If scalar implicature computation is really automatic and not effortful to L2 learners, then our data should show comparable proportions of pragmatic interpretations among low and high proficiency participants. However, this was not the case. Our data provided evidence that low-proficiency participants are less sensitive to the infelicity embedded in under-informative sentences compared to their competent peers, and thus increased language proficiency in L2 seems to be a strong predictor of scalar implicature comprehension among L2 speakers. Similar empirical evidence that supports this proposition comes from a recent study for Mazzaggio et al. [9] on Italian adult speakers of English tested in a sentence evaluation task with time constraints.

Our results seem to corroborate several L1 studies which support claims made by the Relevance theory and argue that the inferential process is cognitively effortful (e.g., [4–6, 16, 20]). However, they fail to reconcile with previous L2 studies on scalar implicature comprehension (e.g., [42, 46, 47]). This discrepancy might be brought on by the presence of implicit time pressure in our study design. We used an online sentence verification task that required participants to judge the truth-value of under-informative sentences *as quickly as possible*. Our instructions may have likely encouraged a feeling of time pressure that limited participants’ cognitive resources during scalar implicature computation. According to Relevance theory, cognitive resources are necessary for inference evaluation and thus fewer inferences are expected when participants’ resources are rendered limited (e.g., [4, 16, 71]). Almost all previous L2 studies on scalar implicature used offline paradigms whose instructions imposed no time pressure on participants’ cognitive resources [42, 46, 47]; and therefore, the participants possibly had more time to elaborate their reasoning at the response stage that resulted in a pragmatic bias A strong piece of evidence in support of this proposition comes from Bott and Noveck’s Experiment 4 [4], which showed that participants derived fewer inferences when the cognitive resources were rendered limited (*Short* condition), but they were more successful at interpreting the implicature when they were given enough time to draw upon the resources that have at their disposal (*Long* condition) (see also [6, 16, 71], for similar arguments).

We suggest that L2 learners may depend on different types of knowledge in online vs. offline tasks, specifically whether it is implicit or explicit knowledge, respectively [72, 73]. According to Ellis [72], a task would measure participant’s implicit knowledge if the design had the participant placed under pressure to perform in real time and on feel. In contrast, a task would measure explicit knowledge if it allows participants to be completely unpressured and have a high degree of attention on form. Our study had instructions that urged participants to respond as fast as possible, and hence our task design may have tapped more into participants’ implicit knowledge than explicit knowledge. In contrast, previous offline paper-based studies, such as Slabakova’s [42], Dupuy et al.’s [47], and Snape and Hasoi’s [46], were entirely absent of time pressure and therefore the superior pragmatic ability of L2 participants could be a proxy of task-related effects that assessed participant’s explicit linguistic knowledge. If this proposition is true, then this may raise important methodological considerations related to the influence of linguistic task on L2 performance in pragmatics and hence to the validity of results interpretations.

Importantly, as one of our reviewers pointed out, our experiment did not involve a deadline procedure that places specific time limits on a trial-by-trial basis, and therefore our explanation of the time pressure as the underlying cause of the behavioral differences between our online experiment and previous offline paper-based tasks could be at stake. This proposition is possible, but the nature of our experiment’s timed stimuli presentation and the instructions that asked participants to respond *as quickly as possible* likely encouraged our respondents to perform in real time; and hence, they had little opportunity to use their metalinguistic knowledge or make adjustments at the response stage before settling on their final decision. We also speculate that adding time constraints would reduce the rate of pragmatic interpretations among our participants and this would still not weaken our overall argument, viz, the interaction effect between response times to T1_logic and T1_pragmatic and Proficiency.

As discussed in the Introduction, the topic of variability in interpreting scalar implicatures has received an increasing interest in experimental pragmatics [14, 18, 29]. Although several studies have attempted to tease apart the factors responsible for pragmatic (in)tolerance, there is still no verdict on which *characteristic* consistently triggers this inter-individual variability in implicature comprehension. Our study examined how L2 proficiency may modulate scalar implicature comprehension among L2 learners and the results provided preliminary exploratory evidence that individual differences in L2 proficiency correlate with both pragmatic rates and pragmatic processing times. To this effect, we were tempted to further examine the role of ToM in scalar implicature derivation. According to classic models of pragmatics (e.g., [1, 11, 31, 74]), scalar implicature computation involves an assessment of the speaker’s epistemic state. Recent psycholinguistic studies showed evidence in support of this view and revealed that individuals vary in their ability to compute others’ mental perspectives [30, 75]. Given that the engagement in ToM abilities are variable across individuals, recent studies on scalar implicatures suggest that those with poor ToM abilities are less likely to compute pragmatic inferences and they tend to be more literal in their interpretations of under-informative sentences [29, 37, 76]. Our results provided evidence in support of this view and showed that those with stronger ToM abilities (as measured by Baron-Cohen’s Social-Skill subscale) tended to calculate more scalar inferences than their peers with weak ToM abilities. Interestingly, this effect seems to be only visible among participants with increased L2 proficiency, although the inteaction with Proficiency did not meet our strict significance criterion. This is only an exploratory result and needs to be taken with caution. However, the effect of social skills on implicature derivation did withstand a stringet correction for multiple comparisons (alpha threshold divided by 21) and it remained visible even after controlling for multiple other variables including the ratings of the B5 and SQ-R questionnaires. This evidence corroborates Fairchild and Papafragou’s recent study on the role of ToM in scalar implicature comprehension [29] and similar other L1 studies showing a significant relationship between participants’ socio-pragmatic skills and rate of pragmatic interpretations [17, 25]. Future research should seek to confirm these exploratory findings and may need to determine the degree of interaction between language proficiency and social skills.

## Conclusions

Overall, our study provides new empirical insights into how L2 learners make pragmatic inferences in real time. In our view, and as shown in our experiment, pragmatic comprehension presents cognitive challenges to L2 speakers and, apparently, L2 learners’ success in implicature understanding seems to depend largely on their L2 language proficiency and pragmatic-social skills. However, one limitation of our study is the absence of an L1 control group in our design. Testing both L1 and L2 individuals with the same stimuli and task can therefore add a valuable piece of evidence to this line of research. Instead of using offline tasks, future research may need to use more robust online testing measures, such as the eye-tracking technique [15, 77] and event-related potential [19, 78] that provide stronger insights into the processes involved in scalar implicature comprehension.

## Supporting information

S1 Appendix The categories and exemplars of the truth-value judgment task. (RTF)
