# Supplementary material for: What second-language speakers can tell us about pragmatic processing
Source: PLoS One. 2022 Feb 18;17(2):e0263724. doi: 10.1371/journal.pone.0263724 (PMC8856516; doi:10.1371/journal.pone.0263724)
Supplement: S1 Appendix — (RTF) [file pone.0263724.s001.rtf]

Appendix A. The categories and exemplars of the truth-value judgment task

Category	Insects	Birds	Fruits	Reptiles	Fish	Flowers	


Exemplar	butterflies	eagles	pineapples	snakes	sharks	lavender	
	mosquitoes	owls	strawberries	dinosaurs	salmons	lilies	
	crickets	penguins	watermelons	chameleons	guppies	hibiscuses	
	grasshoppers	parrots	papayas	tortoises	eels	roses	
	ants	sparrows	grapes	lizards	anchovies	orchids	
	caterpillars	ostriches	durians	crocodiles	piranha	jasmines	
	ladybugs	ducks	coconuts	iguanas	stingray	tulips	
	bees	flamingoes	bananas	frogs	tuna	daisies	
	dragonflies	peacocks	mangos	alligators	sardines	rafflesias	
Note: Latin Square design was used to create six lists of sentences so that every exemplar from a category was used only once per list. This list below shows a sample of the test sentences in one experimental session.

1.	Some butterflies are insects
2.	Some birds are eagles
3.	Some pineapples are flowers
4.	All snakes are reptiles
5.	All fish are sharks
6.	All lavender are fruits
7.	Some mosquitoes are insects
8.	Some birds are owls
9.	Some strawberries are flowers
10.	All dinosaurs are reptiles
11.	All fish are salmons
12.	All lilies are fruits
13.	Some cockroaches are insects
14.	Some birds are penguins
15.	Some watermelons are flowers
16.	All chameleons are reptiles
17.	All fish are anchovies
18.	All hibiscuses are fruits
19.	Some grasshoppers are insects
20.	Some birds are parrots
21.	Some papayas are flowers
22.	All tortoises are reptiles
23.	All fish are eels
24.	All roses are fruits
25.	Some ants are insects
26.	Some birds are sparrows
27.	Some grapes are flowers
28.	All lizards are reptiles
29.	All fish are guppies
30.	All orchids are fruits
31.	Some caterpillars are insects
32.	Some birds are ostriches
33.	Some durians are flowers
34.	All crocodiles are reptiles
35.	All fish are piranha
36.	All jasmines are fruits
37.	Some ladybugs are insects
38.	Some birds are ducks
39.	Some coconuts are flowers
40.	All iguanas are reptiles
41.	All fish are stingrays
42.	All tulips are fruits
43.	Some bees are insects
44.	Some birds are flamingos
45.	Some bananas are flowers
46.	All frogs are reptiles
47.	All fish are tuna
48.	All daisies are fruits
49.	Some dragonflies are insects
50.	Some birds are peacocks
51.	Some mangos are flowers
52.	All alligators are reptiles
53.	All fish are sardines
54.	All rafflesias are fruits
